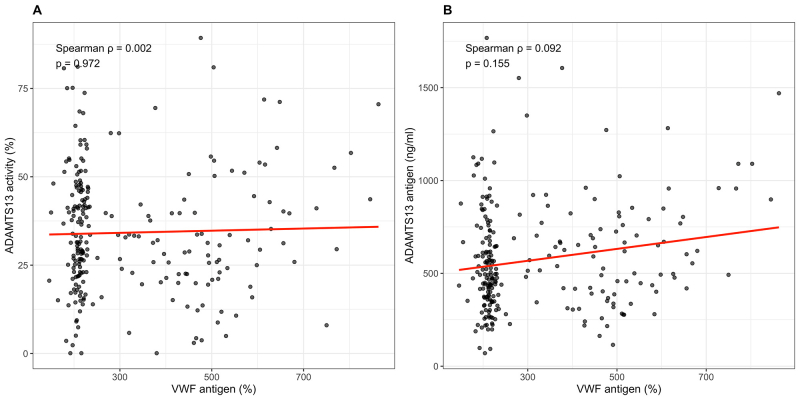# Prognostic value of von Willebrand factor and ADAMTS-13 in patients with sepsis-induced coagulopathy

**DOI:** 10.1016/j.rpth.2026.106843

**Published:** 2026-07-14

**Authors:** Feng Zhang, Yuting Li, Yanhua Li, Dong Zhang

**Affiliations:** The First Hospital of Jilin University, Changchun, China

**Keywords:** ADAMTS-13, prognosis, sepsis, sepsis-induced coagulopathy, von Willebrand factor

## Abstract

**Background:**

The von Willebrand factor (VWF)– a disintegrin and metalloprotease with thrombospondin type 1 repeats, member 13 (ADAMTS-13) axis may contribute to microvascular thrombosis and poor outcomes in sepsis-induced coagulopathy (SIC).

**Objectives:**

The aim of this study was to characterize the VWF–ADAMTS-13 axis in SIC and evaluate whether VWF/ADAMTS-13 ratios are associated with SIC diagnosis and 28-day mortality.

**Methods:**

In this prospective cohort study, 240 adult patients in the intensive care unit meeting Sepsis-3 criteria were consecutively enrolled at the First Hospital of Jilin University from February to October 2025. VWF antigen, factor VIII, ADAMTS-13 activity, and ADAMTS-13 antigen were measured within 24 hours of enrollment. VWF/ADAMTS-13 activity and antigen ratios were calculated. International Society on Thrombosis and Haemostasis SIC and overt disseminated intravascular coagulation scores were derived. The primary outcome was 28-day all-cause mortality. Associations were assessed using multivariable logistic regression, receiver operating characteristic curve analysis, and Kaplan–Meier survival analysis.

**Results:**

Overall, 152 patients (63.3%) met SIC criteria. Compared with non-SIC patients, SIC patients had lower ADAMTS-13 activity, higher VWF/ADAMTS-13 activity and antigen ratios, and higher 28-day mortality. After exclusion of variables included in the SIC score, VWF–ADAMTS-13 biomarkers were not independently associated with SIC diagnosis. Among patients with SIC, nonsurvivors had lower ADAMTS-13 activity and antigen levels but higher VWF/ADAMTS-13 ratios than survivors. In adjusted models, the VWF/ADAMTS-13 activity and antigen ratios were independently associated with 28-day mortality, with modest discriminatory ability (areas under the curve of 0.66 and 0.65, respectively). Mortality increased across VWF/ADAMTS-13 activity ratio quartiles.

**Conclusion:**

VWF/ADAMTS-13 ratios are not independent diagnostic markers for SIC but are independently associated with 28-day mortality in established SIC.

## Essentials

• Sepsis can disturb blood clotting and damage small blood vessels.
• This study included 240 adult patients in the intensive care unit with sepsis.
• A higher VWF/ADAMTS-13 ratio was linked to higher 28-day mortality.
• The ratio may help identify high-risk patients with sepsis-induced coagulopathy.

## 1 Introduction

In the context of the Sepsis-3 definition [1], Iba et al. [2] proposed the diagnostic criteria and scoring system for sepsis-induced coagulopathy (SIC), which is derived from platelet count (PLT), prothrombin time ratio, and the Sequential Organ Failure Assessment (SOFA) score. This scoring system has been shown to predict 28-day mortality more accurately than conventional disseminated intravascular coagulation (DIC) scores. The establishment of the SIC score underscores the critical role of coagulation disorders in the prognosis of sepsis. However, because the SIC score mainly relies on conventional coagulation parameters, substantial heterogeneity in clinical outcomes persists among patients who meet the diagnostic threshold. This suggests that there remains room for improvement in mortality risk stratification within the current scoring framework.

von Willebrand factor (VWF) is a large multimeric glycoprotein synthesized by endothelial cells and megakaryocytes and circulates in plasma in the form of multimers of varying sizes [3]. Upon vascular injury or endothelial activation, VWF binds to exposed collagen and promotes platelet adhesion through interaction between its A1 domain and platelet glycoprotein GPIbα. In addition, VWF stabilizes coagulation factor VIII (FVIII) in the circulation, thereby initiating and contributing to sustained hemostasis [3,4]. Structural abnormalities or dysregulated multimer processing of VWF may result in bleeding, whereas excessive accumulation of highly adhesive VWF multimers, particularly in the setting of impaired ‘a disintegrin and metalloprotease with thrombospondin type 1 repeats, member 13’ (ADAMTS-13)-mediated cleavage, may contribute to thrombotic microangiopathy [3].

ADAMTS-13 is a plasma metalloprotease predominantly synthesized by hepatic stellate cells and secreted into the circulation in its active form. Its primary physiological function is to cleave ultralarge VWF multimers into smaller, less adhesive forms, thereby limiting VWF-mediated platelet adhesion [5]. ADAMTS-13 activity reflects its functional capacity to cleave VWF, whereas ADAMTS-13:Ag represents the quantitative plasma level of the protein. Deficiency of ADAMTS-13 activity leads to impaired processing of VWF, allowing highly adhesive ultralarge multimers to persist on the microvascular endothelium and promote the formation of platelet-rich microvascular thrombi [5]. In the setting of ADAMTS-13 insufficiency, uncleaved VWF may form web-like string structures within the microvasculature, initially capturing platelets and subsequently trapping erythrocytes, ultimately resulting in obstructive microthrombi and profound alterations in local microcirculatory hemodynamics [6]. Unlike D-dimer, a biomarker that primarily reflects fibrin formation and degradation in large-vessel thrombosis [7], VWF-driven microthrombi contain relatively limited amounts of fibrin, rendering D-dimer an insensitive indicator of microthrombotic burden [8]. The VWF/ADAMTS-13 ratio may serve as a composite marker reflecting the imbalance between VWF release and ADAMTS-13-mediated regulation.

Imbalance of the VWF–ADAMTS-13 axis has been repeatedly reported in thrombotic thrombocytopenic purpura [9], COVID-19 [[10], [11], [12]], and DIC [13] and has been closely associated with disease severity and adverse outcomes. In recent years, increasing attention has also been given to abnormalities of this axis in patients with sepsis [14,15]. However, unlike the aforementioned conditions, which are often driven by relatively well-defined thrombotic mechanisms or single etiologies, SIC represents a complex syndrome arising in the context of sepsis and is driven by the interplay of inflammation, endothelial dysfunction, and activation of the coagulation system [16]. Within this specific clinical setting, whether the VWF–ADAMTS-13 axis retains independent diagnostic relevance and prognostic value remains insufficiently supported by systematic prospective evidence.

Therefore, this prospective observational cohort study aimed to characterize early alterations in VWF, ADAMTS-13, and their ratio in patients with SIC and to explore the potential value of these parameters in the diagnosis of SIC and in prognostic risk stratification.

## 2 Methods

### 2.1 Study population

This study was a prospective observational cohort study. From February 2025 through October 2025, consecutive patients with sepsis in the intensive care unit (ICU) of the First Hospital of Jilin University, Changchun, China who met the eligibility criteria were enrolled. All patients fulfilled the Sepsis-3 diagnostic criteria (SOFA score ≥2 points) [1]. Exclusion criteria were: (1) age <18 years; (2) pregnancy or lactation; (3) liver cirrhosis or severe hepatic dysfunction, because liver disease itself may cause substantial coagulation abnormalities that are difficult to distinguish from SIC; (4) a history of hematologic disorders known to affect the VWF–ADAMTS-13 axis or coagulation function, including von Willebrand disease, thrombotic thrombocytopenic purpura, or other thrombotic microangiopathies; and (5) a history of immune-related diseases that may cause thrombosis or coagulation abnormalities, such as antiphospholipid syndrome.

Baseline demographic and clinical data were prospectively collected, including age, sex, body mass index (BMI), comorbidities, infection sites, and disease-related scores. Disease severity was evaluated within 24 hours after enrollment using Acute Physiology and Chronic Health Evaluation II (APACHE II) and SOFA scores, and laboratory parameters related to coagulation and organ function, including PLT and international normalized ratio (INR), were recorded. Plasma levels of VWF antigen (VWF:Ag), FVIII, and ADAMTS-13 activity and antigen were measured. International Society on Thrombosis and Haemostasis (ISTH) SIC and ISTH overt DIC [17] scores were calculated based on clinical and laboratory data obtained during the same period.

### 2.2 Sample collection and processing

Blood samples were obtained from all eligible patients within 24 hours after enrollment. Samples were collected using sodium citrate anticoagulant tubes. After collection, samples were either immediately analyzed or processed and stored according to standardized procedures, depending on the specific assays.

**Figure 1:**
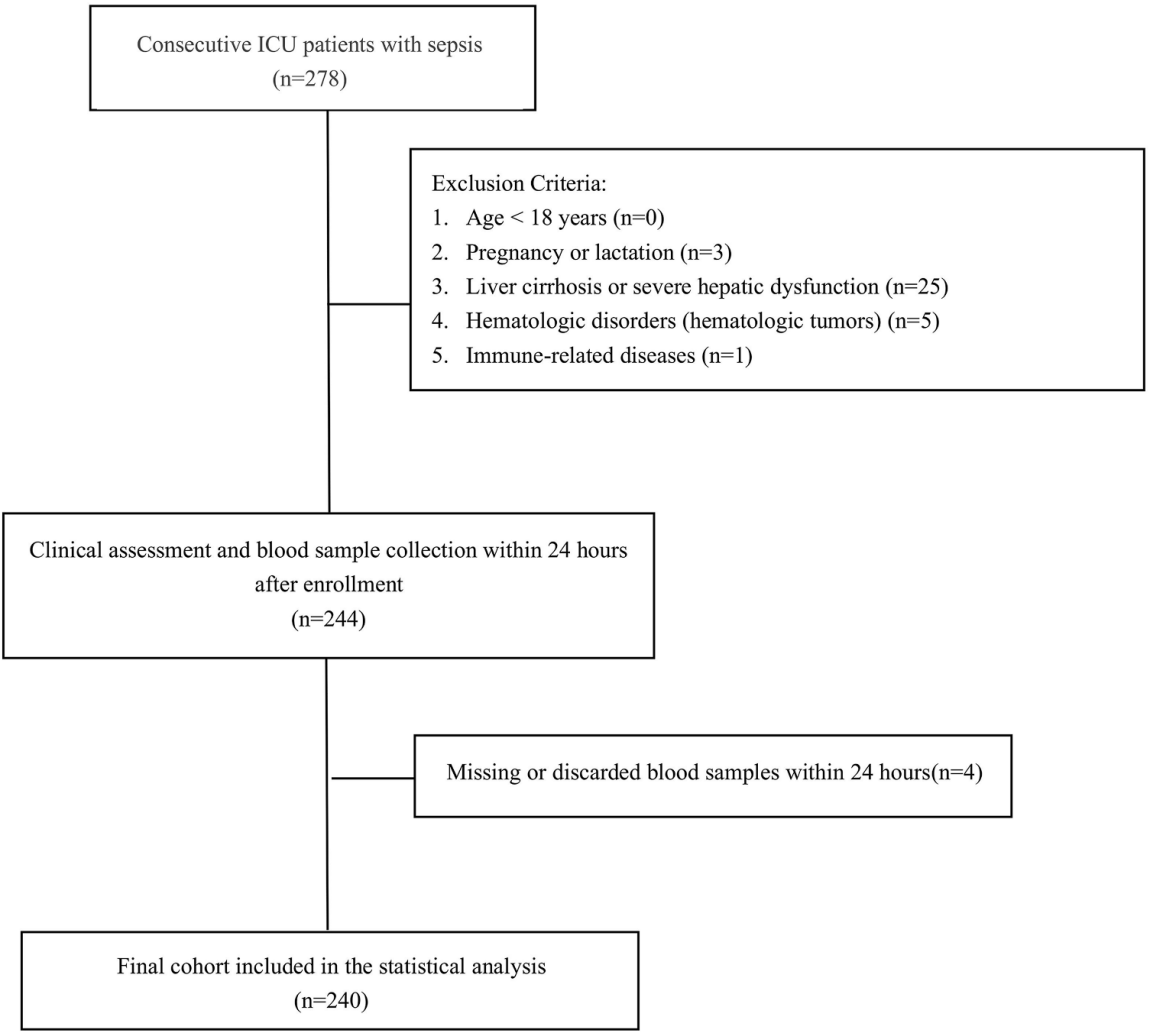
Flowchart of patient enrollment. ICU, intensive care unit.

For external testing, blood samples were centrifuged to separate plasma from blood cells. The plasma was aliquoted into sterile microcentrifuge tubes, with a minimum volume of 200 μL per tube, and immediately stored at −80 °C until analysis. ADAMTS-13 activity was measured using a fluorescence resonance energy transfer assay with a fluorescence microplate reader, while ADAMTS-13:Ag levels were determined by ELISA. VWF:Ag levels were measured in the hospital clinical laboratory using an automated coagulation analyzer (ACL TOP 750, Werfen) based on an immunoturbidimetric assay. All laboratory analyses were performed strictly in accordance with the manufacturers’ instructions and standard operating procedures (Figure 1).

### 2.3 Outcomes

The occurrence of SIC was assessed in all enrolled patients based on laboratory and clinical parameters obtained within 24 hours after enrollment. The primary outcome was 28-day all-cause mortality (28-day mortality). Secondary outcomes included in-hospital mortality, ICU length of stay, and hospital length of stay.

### 2.4 Statistical analysis

Statistical analyses were performed using R software (version 4.4.3). As continuous variables did not meet the assumption of normality, they are presented as medians with IQRs, whereas categorical variables are expressed as counts and percentages. Between-group comparisons were conducted using the Mann–Whitney U-test for continuous variables and the chi-square test or Fisher’s exact test, as appropriate, for categorical variables. The proportion of missing data was low (<10%), and missing values were imputed using single imputation (mean for continuous variables and mode for categorical variables). Univariable and multivariable logistic regression models were applied to evaluate the independent associations between VWF–ADAMTS-13-related biomarkers and SIC as well as 28-day mortality. Multivariable models were adjusted for age, sex, BMI, APACHE II score, and SOFA score. Results are reported as odds ratios (ORs) with corresponding 95% CIs. Receiver operating characteristic curve analysis was performed to assess the predictive performance of the ratio of VWF to ADAMTS-13 activity (VWF/ADAMTS-13 ratio) and the ratio of VWF to ADAMTS-13:Ag (VWF/ADAMTS-13:Ag ratio) for 28-day mortality, and areas under the curve (AUCs) were compared using the DeLong test. Kaplan–Meier survival analysis with the log-rank test was used to compare survival differences among quartiles of the VWF/ADAMTS-13 ratio. All statistical tests were two-sided, and a *P* value < .05 was considered statistically significant.

### 2.5 Ethics approval

This study was reviewed and approved by the Ethics Committee of the First Hospital of Jilin University (approval number: (2022) 2022–013). The study was conducted in accordance with the principles of the Declaration of Helsinki. Written informed consent was obtained from all patients or their legally authorized representatives prior to participation.

## 3 Results

### 3.1 Baseline characteristics

A total of 240 patients with sepsis were included in this study, of whom 152 (63.3%) met the diagnostic criteria for SIC (Table 1). There were no significant differences between the SIC and non-SIC groups with respect to age, sex, BMI, APACHE II score, comorbidities, or sites of infection (all *P* > .05). Compared with the non-SIC group, patients with SIC had significantly higher SOFA scores (8.50 [IQR: 6.50, 11.00] vs 7.00 [IQR: 5.00, 9.00]; *P* < .001), ISTH SIC scores (4.00 [IQR: 4.00, 5.00) vs 3.00 [IQR: 2.00, 3.00]; *P* < .001), and ISTH overt DIC scores (4.00 [IQR: 4.00, 5.00] vs 3.00 [IQR: 2.00, 3.00]; *P* < .001). In addition, platelet counts were significantly lower (108.00 [IQR: 57.50, 170.00] × 10^9^/L vs 202.50 [IQR: 159.00, 271.00] × 10^9^/L; *P* < .001), whereas INR was significantly higher (1.49 [IQR: 1.32, 1.71] vs 1.19 [IQR: 1.11, 1.29]; *P* < .001) in the SIC group. Among coagulation-related variables not included in the SIC diagnostic criteria, VWF:Ag and FVIII levels did not differ significantly between the 2 groups (both *P* > .05), whereas the VWF/FVIII ratio was significantly higher in patients with SIC (1.08 [IQR: 0.79, 1.76] vs 0.98 [IQR: 0.66, 1.33]; *P* = .03]. Patients with SIC also exhibited significantly lower ADAMTS-13 activity (29.44 [IQR: 20.99, 41.78] vs 36.27 [IQR: 24.74, 46.94]; *P* = .03), while ADAMTS-13:Ag levels were lower but not significantly different between groups (496.00 [IQR: 372.50, 667.00] ng/mL vs 562.00 [IQR: 377.50, 765.00] ng/mL; *P* = .17). Accordingly, both the VWF/ADAMTS-13 ratio (9.96 [IQR: 5.81, 17.30] vs 7.28 [IQR: 4.90, 11.70]; *P* = .004) and the VWF/ADAMTS-13:Ag ratio (0.62 [IQR: 0.40, 0.90] vs 0.46 [IQR: 0.31, 0.70]; *P* = .008) were significantly higher in the SIC group. Regarding clinical outcomes, 28-day mortality was significantly higher in the SIC group than in the non-SIC group (40 of 152 patients [26%] vs 12 of 88 patients [14%]; *P* = .03). In-hospital mortality was numerically higher in the SIC group (36 of 152 patients [24%] vs 12 of 88 patients [14%]), although the difference did not reach statistical significance (*P* = .07). ICU length of stay and hospital length of stay did not differ significantly between the 2 groups (both *P* > .05).

**Table 1:** Baseline characteristics of the overall cohort and according to SIC status.

Characteristics	Sepsis (*n* = 240)	SIC (*n* = 152)	Non-SIC (*n* = 88)	*P*
Age (y)	67.00 (57.00, 75.00)	65.50 (57.00, 75.00)	69.00 (60.50, 75.00)	.23
Male	142 (59%)	93 (61%)	49 (56%)	.48
BMI (kg/m^2^)	23.51 (21.24, 25.39)	23.51 (21.26, 25.39)	23.51 (20.81, 25.41)	.66
Comorbidities				
Hypertension	72 (30%)	46 (30%)	26 (30%)	>.99
Diabetes mellitus	52 (22%)	33 (22%)	19 (22%)	>.99
Coronary artery disease	31 (13%)	19 (13%)	12 (14%)	.96
Cerebrovascular disease	31 (13%)	21 (14%)	10 (11%)	.73
Chronic kidney disease	13 (5.4%)	10 (6.6%)	3 (3.4%)	.38
Chronic pulmonary disease	2 (0.8%)	1 (0.7%)	1 (1.1%)	>.99
Malignancy	21 (8.8%)	15 (9.9%)	6 (6.8%)	.57
Others	23 (9.6%)	15 (9.9%)	8 (9.1%)	>.99
None	97 (40%)	58 (38%)	39 (44%)	.42
Infection				
Pulmonary	37 (15%)	23 (15%)	14 (16%)	>.99
Intra-abdominal	170 (71%)	111 (73%)	59 (67%)	.40
Urinary tract	10 (4.2%)	7 (4.6%)	3 (3.4%)	.75
Skin and soft tissue	19 (7.9%)	8 (5.3%)	11 (13%)	.08
Central nervous system	1 (0.4%)	1 (0.7%)	0 (0%)	>.99
Other or unknown	8 (3.3%)	6 (3.9%)	2 (2.3%)	.71
APACHE II	16.00 (12.00, 20.00)	16.00 (12.00, 20.50)	15.00 (11.00, 19.00)	.21
SOFA	8.00 (6.00, 10.50)	8.50 (6.50, 11.00)	7.00 (5.00, 9.00)	<.001
ISTH SIC score	4.00 (3.00, 5.00)	4.00 (4.00, 5.00)	3.00 (2.00, 3.00)	<.001
ISTH overt DIC score	3.00 (3.00, 4.00)	4.00 (4.00, 5.00)	3.00 (2.00, 3.00)	<.001
PLT (10^9^/L)	151.50 (89.00, 226.00)	108.00 (57.50, 170.00)	202.50 (159.00, 271.00)	<.001
INR	1.37 (1.19, 1.57)	1.49 (1.32, 1.71)	1.19 (1.11, 1.29)	<.001
VWF:Ag (%)	221.50 (208.50, 444.50)	221.00 (208.00, 468.50)	222.50 (210.50, 385.50)	.52
FVIII (%)	257.35 (204.70, 342.10)	254.55 (202.50, 333.45)	276.45 (205.10, 364.60)	.18
ADAMTS-13 activity (%)	31.47 (22.54, 44.29)	29.44 (20.99, 41.78)	36.27 (24.74, 46.94)	.03
ADAMTS-13:Ag (ng/mL)	510.50 (374.00, 727.50)	496.00 (372.50, 667.00)	562.00 (377.50, 765.00)	.17
VWF/FVIII ratio	1.05 (0.77, 1.56)	1.08 (0.79, 1.76)	0.98 (0.66, 1.33)	.03
VWF/ADAMTS-13 ratio	9.02 (5.32, 15.65)	9.96 (5.81, 17.30)	7.28 (4.90, 11.70)	.004
VWF/ADAMTS-13:Ag ratio	0.55 (0.37, 0.84)	0.62 (0.40, 0.90)	0.46 (0.31, 0.70)	.008
28-d mortality	52 (22%)	40 (26%)	12 (14%)	.03
In-hospital mortality	48 (20%)	36 (24%)	12 (14%)	.07
ICU LOS (d)	7.00 (4.00, 10.50)	7.00 (4.00, 10.50)	7.00 (4.00, 10.50)	.93
Hospital LOS (d)	10.00 (6.00, 17.00)	10.00 (6.00, 18.00)	9.50 (6.00, 17.00)	.99

Data are presented as *n* (%) or median (Q1, Q3).

ADAMTS-13, a disintegrin and metalloprotease with thrombospondin type 1 repeats, member 13; Ag, antigen; APACHE II, Acute Physiology and Chronic Health Evaluation II; BMI, body mass index; DIC, disseminated intravascular coagulation; FVIII, factor VIII; ICU, intensive care unit; INR, international normalized ratio; ISTH, International Society on Thrombosis and Haemostasis; LOS, length of stay; PLT, platelets; SIC, sepsis-induced coagulopathy; SOFA, Sequential Organ Failure Assessment; VWF, von Willebrand factor.

**Figure 2:**
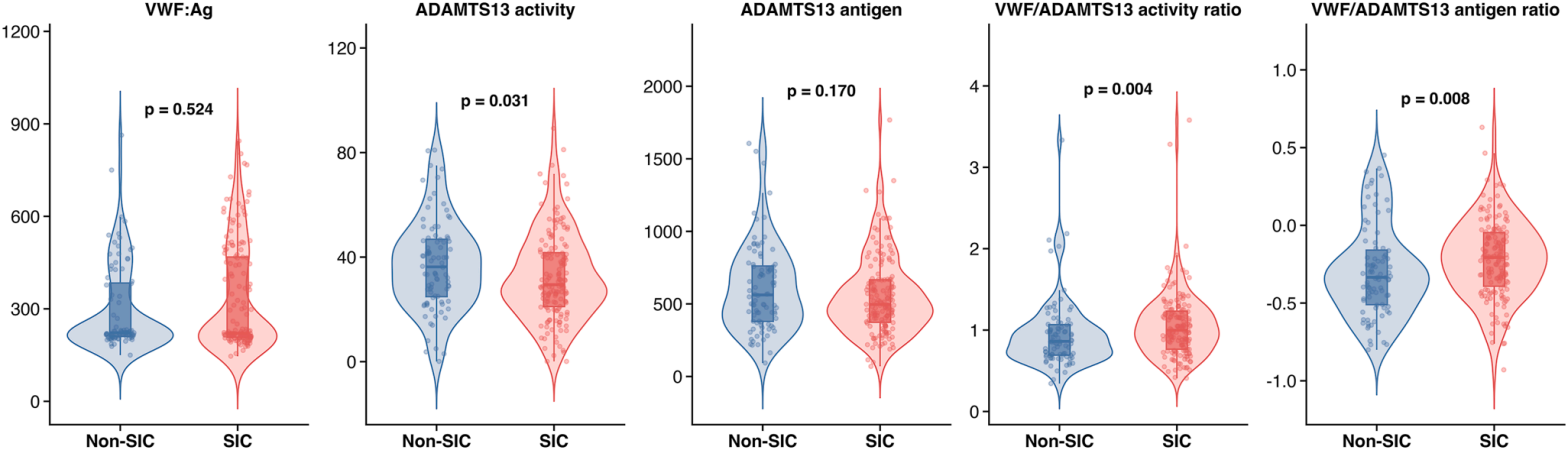
Distributions of VWF–ADAMTS-13-related biomarkers in SIC and non-SIC patients. ADAMTS-13, a disintegrin and metalloprotease with thrombospondin type 1 repeats, member 13; Ag, antigen; SIC, sepsis-induced coagulopathy; VWF, von Willebrand factor.

Figure 2 shows comparisons of VWF–ADAMTS-13-related parameters, including VWF:Ag, ADAMTS-13 activity, ADAMTS-13:Ag, and their ratios, between patients with SIC and those without SIC. Variables that were statistically significant in univariable analyses were entered into the multivariable model (Table 2). Because PLT, INR, and SOFA score are components of the SIC scoring system, they were not entered into the multivariable model to avoid circularity and overadjustment. In addition, the ISTH overt DIC score, which reflects a more advanced stage of coagulation dysfunction, was also excluded from the analysis. Although patients with SIC showed higher VWF/ADAMTS-13 ratios, a higher VWF/FVIII ratio, and lower ADAMTS-13 activity in unadjusted comparisons, none of these biomarkers was independently associated with SIC in the multivariable model. Specifically, the VWF/FVIII ratio was not significantly associated with SIC after adjustment (OR, 1.40; 95% CI, 0.97-2.02; *P* = .07), whereas the VWF/ADAMTS-13 ratio (OR, 1.00; 95% CI, 1.00-1.00; *P* = .89) and the VWF/ADAMTS-13:Ag ratio (OR, 1.03; 95% CI, 0.58-1.85, *P* = .91) were also not statistically significant.

**Table 2:** Multivariable logistic regression for sepsis-induced coagulopathy.

Characteristics	OR	95% CI	*P*
Age	0.99	0.97-1.01	.45
Male	1.27	0.74-2.20	.39
BMI	1.02	0.97-1.07	.46
VWF/FVIII ratio	1.40	0.97-2.02	.07
VWF/ADAMTS-13 ratio	1.00	1.00-1.00	.89
VWF/ADAMTS-13:Ag ratio	1.03	0.58-1.85	.91

ADAMTS-13, a disintegrin and metalloprotease with thrombospondin type 1 repeats, member 13; Ag, antigen; BMI, body mass index; FVIII, factor VIII; OR, odds ratio; VWF, von Willebrand factor.

Among patients with SIC, comparisons according to 28-day mortality showed that nonsurvivors had a significantly lower BMI than survivors (23.09 [IQR: 19.42, 24.17] kg/m^2^ vs 23.51 [IQR: 21.98, 25.87] kg/m^2^; *P* = .02]. Nonsurvivors also had greater disease severity, as reflected by higher APACHE II scores (19.50 [IQR: 14.50, 27.00] vs 15.00 [IQR: 10.50, 19.00]; *P* < .001) and SOFA scores (10.00 [IQR: 8.00, 12.50] vs 8.00 [IQR: 6.00, 11.00]; *P* = .008].With respect to VWF–ADAMTS-13-axis-related biomarkers, nonsurvivors showed significantly lower ADAMTS-13 activity (23.04 [IQR: 14.34, 34.46] vs 31.47 [IQR: 24.66, 43.85]; *P* = .001] and ADAMTS-13:Ag levels (417.50 [IQR: 323.00, 584.00] ng/mL vs 522.00 [IQR: 417.50, 690.50]; *P* = .009), together with higher VWF/ADAMTS-13 ratios (14.27 [IQR: 8.82, 22.20] vs 8.96 [IQR: 5.53, 15.02]; *P* = .002] and VWF/ADAMTS-13:Ag ratios (0.77 [IQR: 0.49, 1.12] vs 0.57 [IQR: 0.37, 0.80]; *P* = .006). Regarding clinical outcomes, nonsurvivors had significantly shorter ICU length of stay (3.00 [IQR: 2.00, 5.50] days vs 8.00 [IQR: 6.00, 12.50]; *P* < .001) and hospital length of stay (4.00 [IQR: 2.00, 10.00] days vs 12.00 [IQR: 7.00, 20.50] days; *P* < .001) than survivors (Table 3).

**Table 3:** Characteristics of patients with SIC according to 28-day mortality

Characteristics	Survivors (*n* = 112)	Nonsurvivors (*n* = 40)	*P* value
Age (years)	65.00 (56.50, 75.00)	66.50 (57.50, 75.00)	.92
Male	71 (63%)	22 (55%)	.46
BMI (kg/m^2^)	23.51 (21.98, 25.87)	23.09 (19.42, 24.17)	.02
ISTH SIC score	4.00 (4.00, 5.00)	5.00 (4.00, 6.00)	.11
ISTH overt DIC score	4.00 (4.00, 5.00)	4.50 (4.00, 5.50)	.14
APACHE II	15.00 (10.50, 19.00)	19.50 (14.50, 27.00)	<.001
SOFA	8.00 (6.00, 11.00)	10.00 (8.00, 12.50)	.008
PLT (10^9^/L)	109.50 (60.00, 190.50)	96.00 (56.00, 156.00)	.17
INR	1.45 (1.31, 1.70)	1.55 (1.37, 1.88)	.12
VWF:Ag (%)	220.00 (207.50, 470.50)	231.50 (210.50, 468.50)	.55
FVIII (%)	272.30 (208.05, 340.30)	242.05 (184.75, 307.90)	.16
VWF/FVIII ratio	1.03 (0.77, 1.64)	1.28 (0.91, 1.87)	.16
ADAMTS-13 activity (%)	31.47 (24.66, 43.85)	23.04 (14.34, 34.46)	.001
ADAMTS-13:Ag (ng/mL)	522.00 (417.50, 690.50)	417.50 (323.00, 584.00)	.009
VWF/ADAMTS-13 ratio	8.96 (5.53, 15.02)	14.27 (8.82, 22.20)	.002
VWF/ADAMTS-13:Ag ratio	0.57 (0.37, 0.80)	0.77 (0.49, 1.12)	.006
ICU LOS (d)	8.00 (6.00, 12.50)	3.00 (2.00, 5.50)	<.001
Hospital LOS (d)	12.00 (7.00, 20.50)	4.00 (2.00, 10.00)	<.001

Data are presented as *n* (%) or median (Q1, Q3).

ADAMTS-13, a disintegrin and metalloprotease with thrombospondin type 1 repeats, member 13; Ag, antigen; APACHE II, Acute Physiology and Chronic Health Evaluation II; BMI, body mass index; DIC, disseminated intravascular coagulation; FVIII, factor VIII; ICU, intensive care unit; INR, international normalized ratio; ISTH, International Society on Thrombosis and Haemostasis; LOS, length of stay; PLT, platelets; SIC, sepsis-induced coagulopathy; SOFA, Sequential Organ Failure Assessment; VWF, von Willebrand factor.

For the analysis of 28-day mortality in patients with SIC, variables that were statistically significant in univariable analyses (BMI, APACHE II score, and SOFA score), together with age and sex, were included as covariates. Given the potential collinearity among ADAMTS-13-related variables and their ratios, 4 separate multivariable logistic regression models were constructed, each including one biomarker related to the VWF–ADAMTS-13 axis. In univariable analyses, ADAMTS-13 activity and antigen levels were inversely associated with 28-day mortality, whereas log (VWF/ADAMTS-13 ratio) and log (VWF/ADAMTS-13:Ag ratio) were positively associated with mortality (Table 4). After adjustment for age, sex, BMI, APACHE II score, and SOFA score, ADAMTS-13 activity in model 1 showed no significant independent association with 28-day mortality (OR, 0.98; 95% CI, 0.95-1.00; *P* = .11). In model 2, log (VWF/ADAMTS-13 ratio) remained significantly associated with increased 28-day mortality (OR, 1.67; 95% CI, 1.10-2.67; *P* = .02). In model 3, ADAMTS-13:Ag was no longer significantly associated with mortality (OR, 1.00; 95% CI, 1.00-1.00; *P* = .14), whereas in model 4, log (VWF/ADAMTS-13:Ag ratio) remained independently associated with increased 28-day mortality (OR, 2.44; 95% CI, 1.12-5.65; *P* = .03) (Table 4).

**Table 4:** Multivariable logistic regression for 28-day mortality in SIC patients.

Characteristics	OR	95% CI	*P*
ADAMTS-13 activity (%)	0.96	0.93-0.99	.003
ADAMTS-13:Ag (ng/mL)	1.00	1.00-1.00	.01
Log (VWF/ADAMTS-13 ratio)	1.73	1.17-2.73	.01
Log (VWF/ADAMTS-13:Ag ratio)	2.28	1.20-4.54	.02
Model 1	0.98	0.95-1.00	.11
Model 2	1.67	1.10-2.67	.02
Model 3	1.00	1.00-1.00	.14
Model 4	2.44	1.12-5.65	.03

Models 1-4 are adjusted logistic regression models, and the reported OR for each model corresponds to the primary biomarker entered into that model. Model 1: Adjusted for age, sex, BMI, APACHE II score, and SOFA score, with ADAMTS-13 activity as the primary variable. Model 2: Adjusted for age, sex, BMI, APACHE II score, and SOFA score, with log (VWF/ADAMTS-13 ratio) as the primary variable. Model 3: Adjusted for age, sex, BMI, APACHE II score, and SOFA score, with ADAMTS-13: Ag as the primary variable. Model 4: Adjusted for age, sex, BMI, APACHE II score, and SOFA score, with log (VWF/ADAMTS-13: Ag ratio) as the primary variable.

ADAMTS-13, a disintegrin and metalloprotease with thrombospondin type 1 repeats, member 13; Ag, antigen; APACHE II, Acute Physiology and Chronic Health Evaluation II; BMI, body mass index; OR, odds ratio; SOFA, Sequential Organ Failure Assessment; VWF, von Willebrand factor.

**Figure 3:**
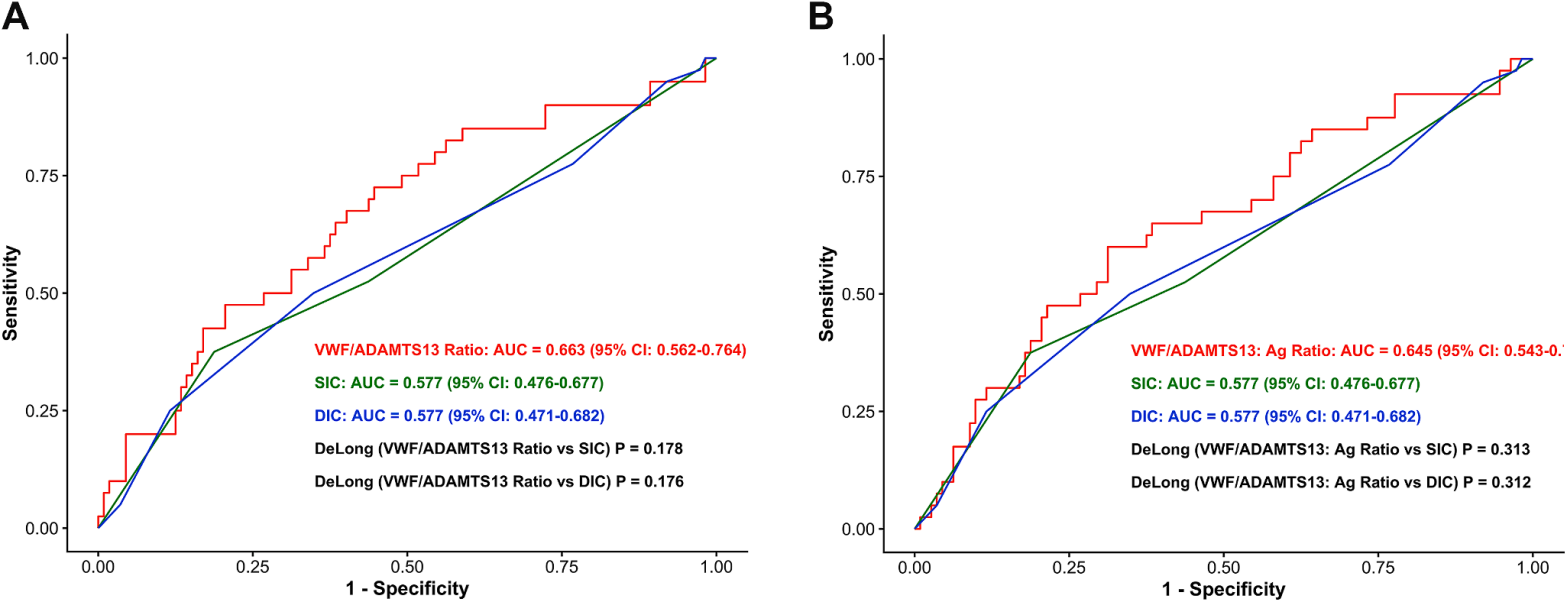
Receiver operating characteristic curve analysis of the VWF/ADAMTS-13 ratio (A), VWF/ADAMTS-13:Ag ratio (B), SIC score, and DIC score for predicting 28-day mortality in patients with SIC. ADAMTS-13, a disintegrin and metalloprotease with thrombospondin type 1 repeats, member 13; Ag, antigen; AUC, area under the curve; DIC, disseminated intravascular coagulation; SIC, sepsis-induced coagulopathy; VWF, von Willebrand factor.

Receiver operating characteristic curve analyses were performed to evaluate the ability of the VWF/ADAMTS-13 activity and antigen ratios to predict 28-day mortality in patients with SIC and to compare their performance with the SIC and DIC scores (Figure 3A, B). As shown in Figure 3A, the VWF/ADAMTS-13 ratio showed modest discriminatory ability for 28-day mortality, with an AUC of 0.66 (95% CI, 0.56-0.76). This AUC was numerically higher than that of the SIC score (AUC, 0.58; 95% CI, 0.48-0.68) and the DIC score (AUC, 0.58; 95% CI, 0.47-0.68), although pairwise comparisons using the DeLong test did not reach statistical significance (*P* = .18 and *P* = .18, respectively). Similarly, as shown in Figure 3B, the VWF/ADAMTS-13:Ag ratio yielded an AUC of 0.65 (95% CI, 0.54-0.75) for predicting 28-day mortality. Although its AUC was numerically higher than those of the SIC and DIC scores, the differences were not statistically significant by the DeLong test.

**Figure 4:**
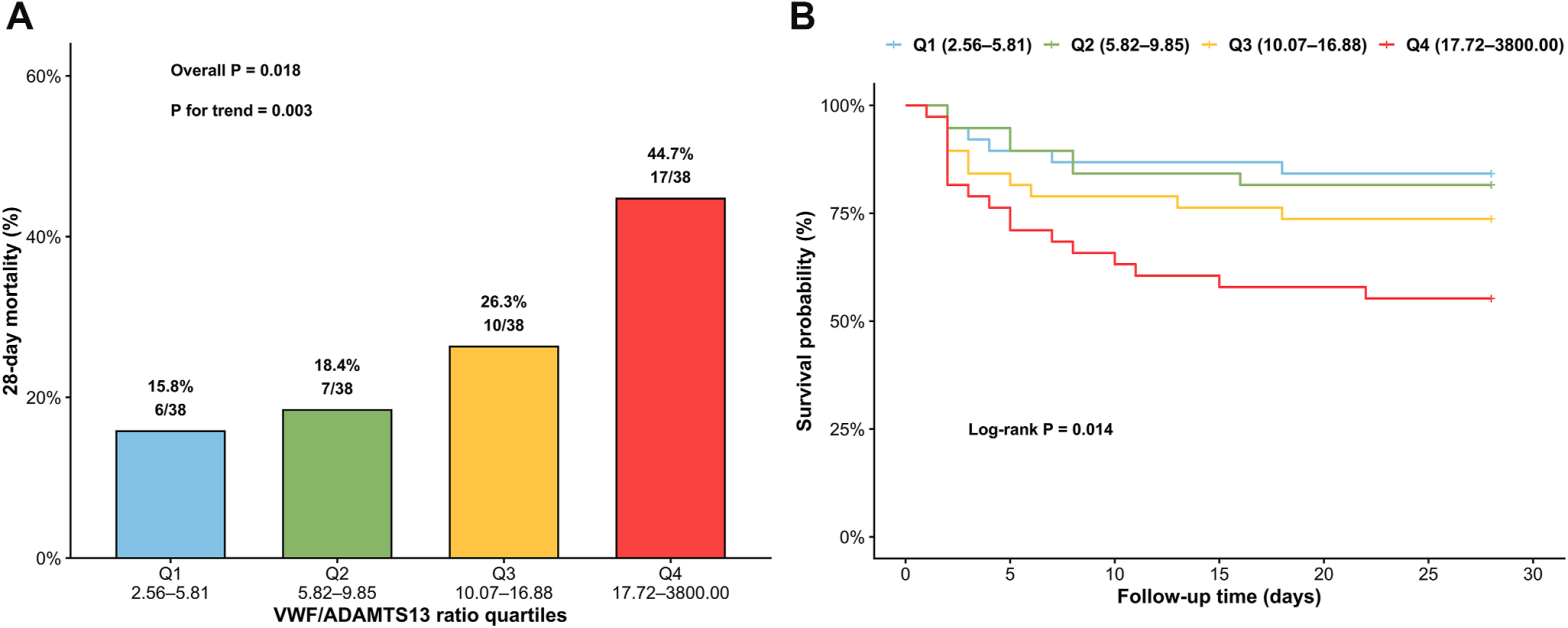
28-day mortality (A) and Kaplan–Meier survival (B) by VWF/ADAMTS-13 ratio quartiles in patients with SIC. ADAMTS-13, a disintegrin and metalloprotease with thrombospondin type 1 repeats, member 13; Q, quartile; SIC, sepsis-induced coagulopathy; VWF, von Willebrand factor.

Figure 4A, B shows 28-day mortality and Kaplan–Meier survival according to quartiles of the VWF/ADAMTS-13 activity ratio among patients with SIC. The quartile ranges of the VWF/ADAMTS-13 ratio were as follows: Q1, 2.56 to 5.81; Q2, 5.82 to 9.85; Q3, 10.07 to 16.88; and Q4, ≥17.72. The bar chart showed an overall increase in 28-day mortality across quartiles, with mortality rates of 15.8%, 18.4%, 26.3%, and 44.7% from Q1 to Q4, respectively. The overall difference among quartiles was statistically significant (χ^2^ = 10.04, *P* = .02), and a significant linear trend was observed (*P* for trend = .003). Consistent with these findings, Kaplan–Meier analysis showed significantly different survival across quartile groups (log-rank *P* = .01). Patients in the highest quartile had the lowest survival probability, whereas those in the lowest quartile had the highest survival probability.

**Figure 5:**
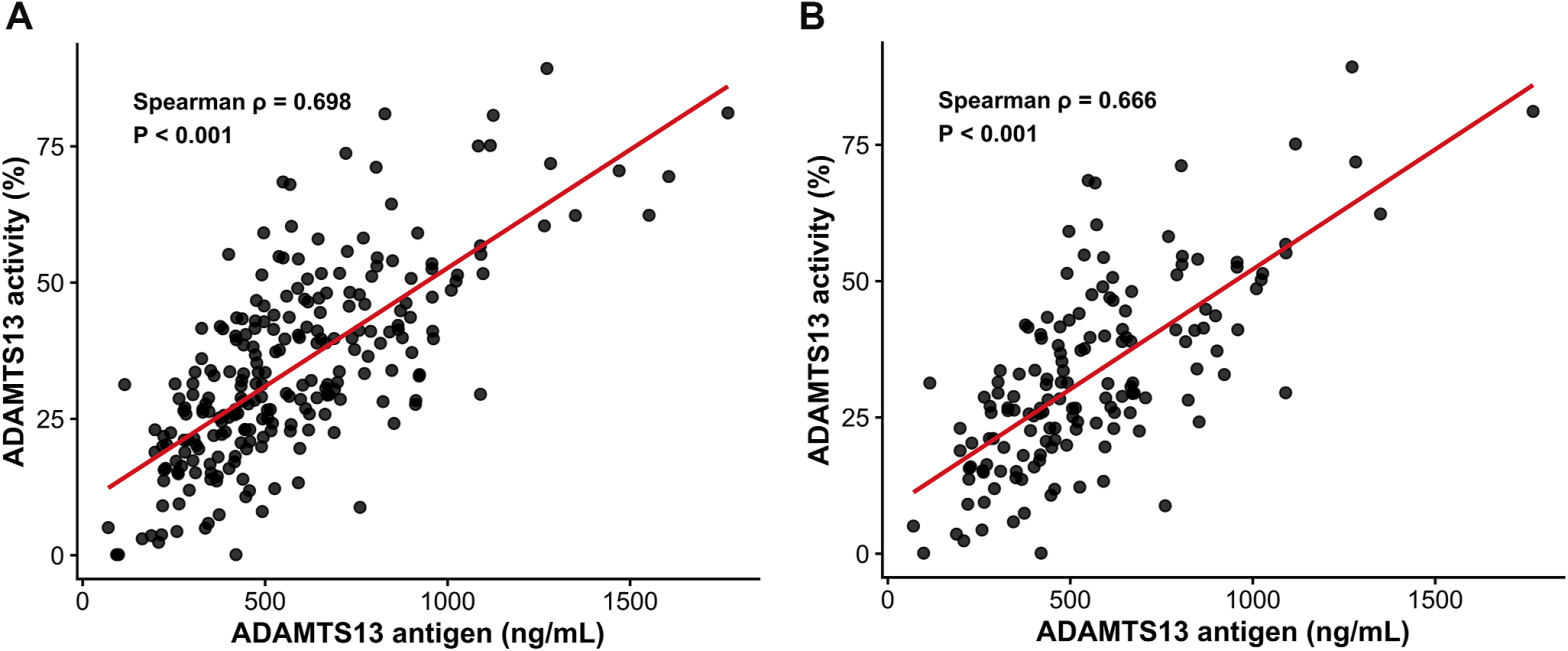
Correlation between ADAMTS-13 antigen and activity in patients with sepsis (A) and sepsis-induced coagulopathy (B). ADAMTS-13, a disintegrin and metalloprotease with thrombospondin type 1 repeats, member 13.

Figure 5A, B illustrates the correlation between ADAMTS-13:Ag levels and ADAMTS-13 activity. In the overall sepsis cohort (Figure 5A), ADAMTS-13:Ag levels were positively correlated with ADAMTS-13 activity (Spearman ρ = 0.70, *P* < .001). A similar significant positive correlation was also observed among patients with SIC (Figure 5B) (Spearman ρ = 0.67, *P* < .001).

## 4 Discussion

This prospective cohort study systematically evaluated alterations of the VWF–ADAMTS-13 axis in patients with SIC and their association with 28-day mortality. We observed a prevalent imbalance characterized by elevated VWF levels concomitant with reduced ADAMTS-13 activity and antigen levels in patients with sepsis, which differs from the findings reported by Fernández et al. [18], who described markedly increased VWF levels with largely preserved ADAMTS-13 activity. In the present study, although ADAMTS-13 activity and antigen levels were inversely associated with 28-day mortality in univariable analyses, these associations were no longer independent after adjustment for age, sex, BMI, APACHE II score, and SOFA score. In contrast, both the VWF/ADAMTS-13 activity ratio and the VWF/ADAMTS-13:Ag ratio remained independently associated with 28-day mortality in multivariable models. Moreover, patients with higher VWF/ADAMTS-13 activity ratios had higher mortality rates and lower survival probability across quartiles. These findings suggest that the relative imbalance of the VWF–ADAMTS-13 axis, rather than the absolute level of any single marker, more comprehensively reflects the dysregulation between VWF release and ADAMTS-13-mediated regulation and is closely associated with adverse outcomes in patients with SIC.

Previous studies in patients with COVID-19, DIC, and other infection-related conditions have reported associations between VWF–ADAMTS-13 imbalance, disease severity, and poor clinical outcomes, with ratio-based indices often showing stronger prognostic associations than individual biomarkers [[11], [12], [13]]. Our study extends these observations to the SIC population and demonstrates an independent association between the VWF/ADAMTS-13 ratio and mortality risk in this prospective cohort. Importantly, although VWF/ADAMTS-13 ratios showed modest discriminatory ability for 28-day mortality and had numerically higher AUCs than SIC and DIC scores, these differences were not statistically significant by the DeLong test. Therefore, the present findings do not support statistical superiority of the VWF/ADAMTS-13 ratio over established SIC or DIC scores. Instead, the ratio may provide complementary prognostic information by reflecting endothelial–coagulation imbalance that is not fully captured by conventional coagulation scores. Although the ratio was significantly associated with SIC occurrence in univariable analysis, this association was attenuated after multivariable adjustment, indicating that the VWF–ADAMTS-13 axis may primarily reflect disease severity and prognostic risk rather than determine whether the diagnostic threshold for SIC is met. Accordingly, the VWF–ADAMTS-13 axis appears to be more suitable as a prognostic stratification marker than as a diagnostic biomarker.

Notably, ADAMTS-13:Ag levels were significantly and positively correlated with ADAMTS-13 activity, but their patterns of change were not entirely consistent in our study. In the comparison between SIC and non-SIC patients, ADAMTS-13 activity was significantly reduced, whereas antigen levels showed only a downward trend, suggesting that within the same sampling window, ADAMTS-13 activity may more readily reflect sepsis-related coagulopathic disturbances than antigen levels. In contrast, among patients with SIC, nonsurvivors exhibited lower levels of both ADAMTS-13 activity and antigen, suggesting that in more severe disease, ADAMTS-13 abnormalities may involve not only functional impairment but also a reduction in circulating protein levels. We speculate that this difference may reflect distinct patterns of ADAMTS-13 alteration across different degrees of disease severity. Inflammatory conditions may reduce ADAMTS-13 activity through multiple mechanisms, including functional inactivation, degradation, and possibly reduced production or expression, although the relative contribution of these mechanisms remains unclear [[19], [20], [21]]. Additionally, free hemoglobin released during intravascular hemolysis—which may accompany sepsis-associated erythrocyte injury—can directly inhibit ADAMTS-13 activity by binding to the A2 domain of VWF and competitively blocking the ADAMTS-13 cleavage site, thereby further amplifying the VWF–ADAMTS-13 imbalance [22,23]. Whether hemolysis-driven inhibition of ADAMTS-13 contributed to the elevated VWF/ADAMTS-13 ratio observed in our SIC cohort warrants further investigation.

From a pathophysiological perspective, systemic inflammation in sepsis leads to widespread endothelial activation and excessive VWF release, while reduced ADAMTS-13 activity further limits VWF cleavage [15]. The resulting accumulation of highly adhesive VWF multimers promotes platelet adhesion and microvascular thrombosis, exacerbating microcirculatory dysfunction and organ injury. Therefore, an elevated VWF/ADAMTS-13 ratio may be viewed as a composite marker reflecting the severity of endothelial–coagulation imbalance and its association with adverse prognosis. As this study is observational, the underlying mechanisms require further elucidation and validation in multicenter cohorts; nevertheless, our findings indicate that the VWF/ADAMTS-13 ratio has potential prognostic stratification value and may provide complementary information for risk assessment in patients with SIC.

### 4.1 Conclusion

In summary, although biomarkers related to the VWF–ADAMTS-13 axis were not independent diagnostic factors for SIC, the VWF/ADAMTS-13 ratio was independently associated with 28-day mortality among patients with established SIC. Compared with individual VWF or ADAMTS-13 measurements, this ratio may better capture the relative imbalance between VWF release and ADAMTS-13-mediated regulation and may provide complementary information for prognostic risk stratification in patients with SIC.

### 4.2 Limitations

This study has several limitations. First, as a single-center prospective observational study, the generalizability of our findings requires confirmation in larger, multicenter cohorts. The sample size was determined by the number of eligible patients enrolled during the predefined study period rather than by a formal power calculation. In addition, the relatively high proportion of patients with severe infection and intra-abdominal infection may reflect the case mix and disease severity of our ICU population and should be considered when interpreting the representativeness of the findings. Although infection sites were recorded, detailed pathogen-level classification was not systematically available. Second, VWF and ADAMTS-13 levels were measured only once at early enrollment, precluding assessment of their dynamic changes over time and their relationship with disease progression and outcomes. Moreover, VWF was assessed only as VWF:Ag, whereas functional VWF assays and VWF multimer analysis were not performed. Third, hemolysis could not be comprehensively evaluated because lactate dehydrogenase, haptoglobin, cell-free hemoglobin, and peripheral blood smear findings were not available. In addition, anti-ADAMTS-13 antibody testing and detailed medication-level assessment, including calcineurin inhibitor exposure, were not routinely performed. Finally, although key confounding variables were adjusted in multivariable analyses, residual confounding cannot be completely excluded.

## Figures and Tables

**Supplementary Figure S1:**
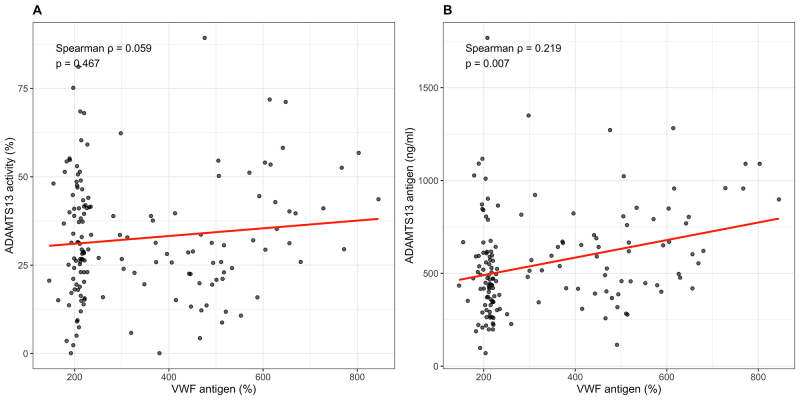


**Supplementary Figure S2:**