# Supplementary material for: Prognostic value of von Willebrand factor and ADAMTS-13 in patients with sepsis-induced coagulopathy
Source: Res Pract Thromb Haemost. 2026 Jul 14;10(5):106843. doi: 10.1016/j.rpth.2026.106843 (PMC13448020; doi:10.1016/j.rpth.2026.106843)
Supplement: Supplementary file 1 [file mmc1.pdf]

委托单位	吉林大学第一医院		
委托人	张凤	联系方式	155 3363 1069
被委托单位	广州知力医学诊断技术有限公司		
项目负责人	血液实验室	联系方式	020-31601995

ADAMTS 13 活性检测报告（共 240 例）

报告日期：2025-11-04

实验结果汇总：

姓名	床号	EP 管编号	入科时间	住院号	活性结果	**方法学**
任玉珍	311	0225311	2025/2/24	12838011	23.03	荧光共振能量转移法
肖庆彬	319	0224319	2025/2/24	12825560	19.9	荧光共振能量转移法
刘长宽	306	0227306	2025/2/26	11636562	25.88	荧光共振能量转移法
周玉珍	104	0227104	2025/2/26	12848119	39.52	荧光共振能量转移法
苏凤琴	302	0227302	2025/2/26	12848467	41.6	荧光共振能量转移法
吕春珍	106	0226106	2025/2/26	12848542	41.42	荧光共振能量转移法

广州知力医学检验实验室
报告专用章

战玉娥	303	0227303	2025/2/27	12848543	39.81	荧光共振能量转移法
王丽梅	121	0302121	2025/3/1	12849569	31.21	荧光共振能量转移法
侯美荣	102	0302102	2025/3/2	12849758	44.52	荧光共振能量转移法
马文忠	305	0306305	2025/3/5	12851241	22.46	荧光共振能量转移法
刘金英	202	0308202	2025/3/7	11620705	48.60	荧光共振能量转移法
李淑英	201	0308201	2025/3/7	12852548	18.89	荧光共振能量转移法
张志武	221	0312221	2025/3/11	12342603	41.38	荧光共振能量转移法
文明	210	0312210	2025/3/11	12854776	36.76	荧光共振能量转移法
陈先超	103	0313103	2025/3/12	12854823	25.65	荧光共振能量转移法
冯桂杰	206	0316206	2025/3/15	12856368	60.41	荧光共振能量转移法
张秀珍	305	0317305	2025/3/16	12803400	25.17	荧光共振能量转移法
代国才	320	250319j320	2025/3/18	12857738	33.66	荧光共振能量转移法
赵志兰	208	0319208	2025/3/19	12805358	28.84	荧光共振能量转移法
张桂兰	107	0321107	2025/3/20	11822152	58.18	荧光共振能量转移法
杜善民	306	0321306	2025/3/20	12858822	11.82	荧光共振能量转移法
许凤芹	211	0320211	2025/3/20	12858873	17.25	荧光共振能量转移法
马艳霞	307	0322307	2025/3/21	12807945	20.29	荧光共振能量转移法
苏中英	115	0324115	2025/3/21	12859792	20.16	荧光共振能量转移法
宋多军	320	250324320	2025/3/22	12860093	38.56	荧光共振能量转移法
高军	319	0325319	2025/3/24	12365545	33.14	荧光共振能量转移法
季生发	320	250325320	2025/3/25	12855501	36.48	荧光共振能量转移法
张淼	220	0327220	2025/3/26	12675458	48.09	荧光共振能量转移法
李斌	304	0327304	2025/3/26	12860040	26.34	荧光共振能量转移法
张永芬	306	0327306	2025/3/26	12862266	31.3	荧光共振能量转移法
邢永海	221	0328221	2025/3/27	12862602	26.76	荧光共振能量转移法

报告专用章

李芳	102	0329102	2025/3/28	12863199	38.2	荧光共振能量转移法
双喜	221	0331221	2025/3/29	12863456	36.06	荧光共振能量转移法
沈洪学	212	0331212	2025/3/30	12863765	31.48	荧光共振能量转移法
齐志红	320	0402J320	2025/4/1	12863253	33.52	荧光共振能量转移法
王春华	218	0402218	2025/4/1	12865003	27.76	荧光共振能量转移法
王维岐	112	0402112	2025/4/2	12399740	21.39	荧光共振能量转移法
康志学	203	0404203	2025/4/3	11971147	58	荧光共振能量转移法
陈玉环	307	0406307	2025/4/4	12862974	16.69	荧光共振能量转移法
腾凤香	110	0406110	2025/4/4	12864547	25.76	荧光共振能量转移法
张连海	203	0406203	2025/4/4	12866035	44.05	荧光共振能量转移法
隋素花	306	250409306	2025/4/7	12859534	45.73	荧光共振能量转移法
郭志才	201	0411201	2025/4/10	12868683	23.04	荧光共振能量转移法
王学政	218	0413218	2025/4/11	11048821	15.07	荧光共振能量转移法
宋希贵	205	0411205	2025/4/11	12864736	21.12	荧光共振能量转移法
窦中花	319	0413319	2025/4/11	12869485	43.54	荧光共振能量转移法
赵志国	115	0415115	2025/4/13	12364666	21.13	荧光共振能量转移法
何贵军	220	0413220	2025/4/13	12825413	26.24	荧光共振能量转移法
张生	311	0416311	2025/4/15	00017501	59.13	荧光共振能量转移法
衡国付	317	0416317	2025/4/15	12871359	46.72	荧光共振能量转移法
陈石	303	0416303	2025/4/15	12871561	32.03	荧光共振能量转移法
马孝臣	208	0417208	2025/4/16	12869203	23	荧光共振能量转移法
马晓艳	113	0416113	2025/4/16	12871403	9.413	荧光共振能量转移法
李淑霞	207	0419207	2025/4/17	12196143	41.83	荧光共振能量转移法
孙洪财	305	0421305	2025/4/19	12873043	31.44	荧光共振能量转移法
王玉艳	203	0421203	2025/4/21	12873722	28.85	荧光共振能量转移法

报告专用章

王俊平	308	0421308	2025/4/21	12874212	53.01	荧光共振能量转移法
包宝喜	311	0424311	2025/4/23	12875228	37.63	荧光共振能量转移法
刘海莉	305	0425305	2025/4/24	12833547	47.52	荧光共振能量转移法
唐玉祥	305	0426305	2025/4/25	12876477	15.64	荧光共振能量转移法
王桂荣	302	0428302	2025/4/27	11666519	29.45	荧光共振能量转移法
兰桂芹	111	0429111	2025/4/27	12874823	68.47	荧光共振能量转移法
付红光	313	0428313	2025/4/27	12877298	51.15	荧光共振能量转移法
汪秀芬	205	0430205	2025/4/29	12875871	54.53	荧光共振能量转移法
陈喜任	202	0430202	2025/4/30	12878178	68.01	荧光共振能量转移法
哈斯	218	0501218	2025/4/30	12878538	39.90	荧光共振能量转移法
刘玉杰	208	0502208	2025/5/1	12878589	41.62	荧光共振能量转移法
张士芹	117	0502117	2025/5/1	12878590	80.96	荧光共振能量转移法
王轩博	320	0504320	2025/5/3	12879008	19.97	荧光共振能量转移法
张凤梅	310	0505310	2025/5/4	12879372	15.93	荧光共振能量转移法
王锡富	109	0506109	2025/5/5	12879689	20.86	荧光共振能量转移法
王全	311	0506311	2025/5/5	12879403	40.95	荧光共振能量转移法
王金	203	0506203	2025/5/6	12879808	28.23	荧光共振能量转移法
田绍金	221	0507221	2025/5/6	12879852	50.66	荧光共振能量转移法
高淑琴	205	0508205	2025/5/7	12880626	55.18	荧光共振能量转移法
郭学会	108	0508108	2025/5/7	12881101	8.003	荧光共振能量转移法
段友发	202	0508202	2025/5/7	12881195	21.82	荧光共振能量转移法
王联海	111	0509111	2025/5/8	11472578	42.83	荧光共振能量转移法
王杰	101	0509101	2025/5/8	12881230	80.69	荧光共振能量转移法
卢占军	203	0510203	2025/5/9	12881233	35.25	荧光共振能量转移法
戴永贵	220	0510220	2025/5/9	12881739	5.839	荧光共振能量转移法

于国平	221	0512221	2025/5/11	11590052	48.22	荧光共振能量转移法
于洪福	102	0512102	2025/5/11	12715603	43.64	荧光共振能量转移法
王凯	218	0512218	2025/5/11	12882480	29.43	荧光共振能量转移法
张丽华	204	0512204	2025/5/11	12882766	39.66	荧光共振能量转移法
潘立财	319	0514319	2025/5/13	12884187	41.14	荧光共振能量转移法
张春华	106	0514106	2025/5/14	12875377	5.06	荧光共振能量转移法
张凤传	119	0516119	2025/5/15	11605382	27.75	荧光共振能量转移法
李殿军	315	0515315	2025/5/15	12884724	28.57	荧光共振能量转移法
乔艳春	121	0516121	2025/5/15	12885141	69.46	荧光共振能量转移法
唐连仲	302	0517302	2025/5/16	12451113	20.61	荧光共振能量转移法
高晓阳	318	0517318	2025/5/16	12884730	29.15	荧光共振能量转移法
李志芹	313	0517313	2025/5/16	12885428	29.06	荧光共振能量转移法
王玉兰	219	250519219	2025/5/17	12832366	9.065	荧光共振能量转移法
李志刚	220	250519220	2025/5/18	12885968	0.1	荧光共振能量转移法
张广才	318	250520318 张广才	2025/5/19	12881730	46.46	荧光共振能量转移法
李淑华	207	0520207	2025/5/19	12886596	25.28	荧光共振能量转移法
郝向文	215	0521215	2025/5/20	11355935	28.71	荧光共振能量转移法
张宇	111	0522111	2025/5/22	12887186	32.86	荧光共振能量转移法
张玉梅	208	0522208	2025/5/22	12888407	55.72	荧光共振能量转移法
董岩	221	0522221	2025/5/22	12888407	31.45	荧光共振能量转移法
柳树文	113	0523113	2025/5/23	12628820	32.06	荧光共振能量转移法
王际广	320	0523320	2025/5/23	12765873	54.33	荧光共振能量转移法
温希君	102	0523102	2025/5/23	12889013	51.42	荧光共振能量转移法
曲冬梅	319	20250528319	2025/5/27	12890343	8.78	荧光共振能量转移法
刘旭东	117	250530117	2025/5/28	12731155	7.423	荧光共振能量转移法

广州知力医学检验实验室 报告专用章

王延平	103	250529103	2025/5/28	12872844	39.74	荧光共振能量转移法
付书英	303	250529303	2025/5/28	12890975	40.49	荧光共振能量转移法
张万春	306	0530306	2025/5/29	12891310	42.98	荧光共振能量转移法
何亚娇	212	0530212	2025/5/29	12891556	43.36	荧光共振能量转移法
王清春	218	0601218	2025/5/31	12892064	15.9	荧光共振能量转移法
代红伟	208	0603208	2025/6/1	12886312	29.67	荧光共振能量转移法
郭庆文	209	0603209	2025/6/1	12892001	41.95	荧光共振能量转移法
徐亚芬	102	0601102	2025/6/1	12892175	51.71	荧光共振能量转移法
张志元	106	250603106	2025/6/1	12892458	19.5	荧光共振能量转移法
李凤云	304	20250606304	2025/6/4	12893859	11.93	荧光共振能量转移法
王旭红	313	20250606313	2025/6/5	12891473	14.93	荧光共振能量转移法
孙淑英	104	0606104	2025/6/5	12893088	14.46	荧光共振能量转移法
张群力	306	20250606306	2025/6/5	12894263	22.78	荧光共振能量转移法
张培琪	103	0607103	2025/6/6	12886137	0.1	荧光共振能量转移法
许国君	113	0607113	2025/6/6	12894781	24.20	荧光共振能量转移法
王延平	116	20250609116	2025/6/7	12872844	3.01	荧光共振能量转移法
包宝龙	217	20250609217	2025/6/7	12895259	51.67	荧光共振能量转移法
张春安	117	20250609117	2025/6/7	12895331	24.47	荧光共振能量转移法
温凤江	219	250610219	2025/6/9	12895882	53.46	荧光共振能量转移法
郑宝石	315	250610315	2025/6/10	12896145	51.65	荧光共振能量转移法
张宝义	306	0614306	2025/6/14	12898571	12.21	荧光共振能量转移法
马贵	118	0616118	2025/6/15	11773263	46.26	荧光共振能量转移法
王纪英	106	0616106	2025/6/15	12899237	89.28	荧光共振能量转移法
逯胜金	107	0616107	2025/6/16	12899238	22.15	荧光共振能量转移法
张建明	208	250618208	2025/6/17	12900536	10.72	荧光共振能量转移法

报告专用章

刘忠山	306	0622306	2025/6/21	12902059	26.9	荧光共振能量转移法
王秀兰	218	0623218	2025/6/22	12902571	70.52	荧光共振能量转移法
马启云	308	0624308	2025/6/23	12899970	2.36	荧光共振能量转移法
刘明友	316	0625316	2025/6/24	12903814	0.1	荧光共振能量转移法
祝福	319	0627319	2025/6/26	12585995	31.7	荧光共振能量转移法
刘凤芝	302	0627302	2025/6/26	12904319	21.95	荧光共振能量转移法
董书有	220	0628220	2025/6/27	12518227	29.38	荧光共振能量转移法
张耀魁	308	0628308	2025/6/27	12905156	59.09	荧光共振能量转移法
李成建	111	0629111	2025/6/28	12905416	26.7	荧光共振能量转移法
邹继刚	204	250701204	2025/6/30	12904202	41.07	荧光共振能量转移法
李永芹	202	250701202	2025/6/30	12905744	17.35	荧光共振能量转移法
李桂珍	319	0701319	2025/7/1	12906487	56.75	荧光共振能量转移法
于珍	206	20250703206	2025/7/2	12907094	37.3	荧光共振能量转移法
尹志强	216	20250703216	2025/7/2	12907141	64.41	荧光共振能量转移法
韩杰	115	250704115	2025/7/3	12255216	39.71	荧光共振能量转移法
柴永波	309	250704309	2025/7/3	12908078	52.55	荧光共振能量转移法
包力德日高	318	0706318	2025/7/5	12908480	40.37	荧光共振能量转移法
刘洪岩	202	0707202	2025/7/6	12908829	26.5	荧光共振能量转移法
姜春有	117	0708117	2025/7/7	12423866	46.96	荧光共振能量转移法
韩凤侠	110	0709110	2025/7/8	12847711	23.08	荧光共振能量转移法
付玉清	112	20250709112	2025/7/8	12909847	40.2	荧光共振能量转移法
刘福芹	201	20250710201	2025/7/9	12822280	19.49	荧光共振能量转移法
杨国发	120	0711120	2025/7/10	12911290	39.94	荧光共振能量转移法
赵文江	306	0714306	2025/7/13	12892753	71.17	荧光共振能量转移法
赵洪丽	118	20250715118	2025/7/14	12912389	30.62	荧光共振能量转移法

广州知力医学检验实验室
报告专用章

尹凤云	310	250716310	2025/7/15	12913342	54.56	荧光共振能量转移法
张少华	302	0717302	2025/7/16	12914329	45.68	荧光共振能量转移法
陆玉华	320	0718320	2025/7/17	12914776	26.03	荧光共振能量转移法
王守文	304	0720304	2025/7/19	12915380	28.41	荧光共振能量转移法
谢学勤	221	0721221	2025/7/20	12915511	30.46	荧光共振能量转移法
董林	306	250722306	2025/7/21	12916377	48.95	荧光共振能量转移法
张兴龙	207	250722207	2025/7/21	12916599	37.18	荧光共振能量转移法
荆纪元	320	20250724303	2025/7/23	11486900	51.39	荧光共振能量转移法
连桂芝	221	250725221	2025/7/24	12916291	25.91	荧光共振能量转移法
杨继华	101	0725101	2025/7/24	12918213	28.59	荧光共振能量转移法
谢井玲	108	0726108	2025/7/25	12918606	26.64	荧光共振能量转移法
杨永臣	112	0727112	2025/7/26	12404647	18.02	荧光共振能量转移法
徐忠	105	0428111-1	2025/7/26	12918594	60.33	荧光共振能量转移法
朱艳森	113	250729113	2025/7/28	12918920	54.79	荧光共振能量转移法
王淑伟	208	0803208	2025/8/2	12921962	18.89	荧光共振能量转移法
李淑艳	301	2025804301	2025/8/3	12916032	44.84	荧光共振能量转移法
谢跃武	117	250805117	2025/8/4	12834442	33.67	荧光共振能量转移法
刘书琴	221	250805221	2025/8/4	12922581	46.04	荧光共振能量转移法
刘树仁	111	20250806111	2025/8/5	12679536	47.15	荧光共振能量转移法
吴志	308	20250806308	2025/8/5	12923349	33.90	荧光共振能量转移法
李淑琴	303	0802303	2025/8/7	12923812	47.79	荧光共振能量转移法
单洪光	304	0810304	2025/8/9	11621050	24.17	荧光共振能量转移法
李福春	101	0810101	2025/8/9	12924923	41.23	荧光共振能量转移法
马玉芹	306	250812306	2025/8/11	12872533	23.96	荧光共振能量转移法
陈淑清	209	250812209	2025/8/11	12926006	27.67	荧光共振能量转移法

报告专用章

潘丽君	119	250812119	2025/8/11	12926729	31.35	荧光共振能量转移法
邵柱文	221	0813221	2025/8/12	12926734	22.96	荧光共振能量转移法
贾香阁	221	250815221	2025/8/14	11303331	33.87	荧光共振能量转移法
杨威山	304	0817304	2025/8/15	12923855	81.13	荧光共振能量转移法
魏道行	221	0816221	2025/8/16	12928796	41.53	荧光共振能量转移法
张洪国	221	0817221	2025/8/17	12929069	75.07	荧光共振能量转移法
杨艳芳	102	0820102	2025/8/19	12930563	21.63	荧光共振能量转移法
李淑艳	219	0820219	2025/8/19	12930617	17.12	荧光共振能量转移法
李桂荣	315	0821315	2025/8/20	12914072	27.04	荧光共振能量转移法
王希江	202	20250821202	2025/8/20	12930639	54.01	荧光共振能量转移法
王永强	218	0822218	2025/8/21	12929734	37.73	荧光共振能量转移法
何显峰	209	0824209	2025/8/23	12929045	62.36	荧光共振能量转移法
高代岭	202	0824202	2025/8/23	12932055	55.18	荧光共振能量转移法
王献勤	208	0824208	2025/8/23	12932229	71.85	荧光共振能量转移法
李树元	304	0824304	2025/8/23	12932252	41.08	荧光共振能量转移法
徐殿玉	204	0825304	2025/8/24	12565665	22.49	荧光共振能量转移法
陈书媛	103	20250827103	2025/8/26	12931448	47.33	荧光共振能量转移法
高志荣	120	20250828120	2025/8/27	12324945	15.25	荧光共振能量转移法
鲁占山	316	20250828316	2025/8/27	12933924	33.28	荧光共振能量转移法
刘淑香	217	20250828217	2025/8/27	12933928	13.92	荧光共振能量转移法
于海林	220	0831220	2025/8/30	12935322	3.745	荧光共振能量转移法
霍荣华	104	0831104	2025/8/30	12935559	13.61	荧光共振能量转移法
李立明	311	0831311	2025/8/31	12935580	50.77	荧光共振能量转移法
王艳波	303	250902303	2025/9/1	12936352	50.24	荧光共振能量转移法
王志萍	319	20250904319	2025/9/3	12937516	33.55	荧光共振能量转移法

报告专用章

刘桂艳	209	0905209	2025/9/4	12933031	4.966	荧光共振能量转移法
董昕卓	306	0905306	2025/9/5	00806280	3.581	荧光共振能量转移法
刘会君	109	0906109	2025/9/5	12345468	25.85	荧光共振能量转移法
汪佳荣	304	0906304	2025/9/5	12938244	43.64	荧光共振能量转移法
刘书真	320	0909320	2025/9/8	12939003	22.82	荧光共振能量转移法
邱琬玥	113	0909113	2025/9/8	12939647	75.16	荧光共振能量转移法
高雨林	108	20250910108	2025/9/9	12897064	25.12	荧光共振能量转移法
邹万发	111	0914111	2025/9/13	12941576	31.03	荧光共振能量转移法
张兰	117	0914117	2025/9/13	12941808	13.93	荧光共振能量转移法
吴会娟	206	0917206	2025/9/16	12942142	19.59	荧光共振能量转移法
孙中芹	212	0917212	2025/9/16	12943187	18.12	荧光共振能量转移法
李龙蛟	101	0919101	2025/9/19	12944405	32.95	荧光共振能量转移法
李凤	306	2025922306	2025/9/21	11800557	29.52	荧光共振能量转移法
宋庆	202	250923202	2025/9/22	12945363	25.00	荧光共振能量转移法
耿玉林	319	20250925319	2025/9/24	12946630	38.88	荧光共振能量转移法
朱京里	110	250926110	2025/9/25	12947570	62.31	荧光共振能量转移法
李金	218	0928218	2025/9/27	12937747	31.32	荧光共振能量转移法
姜连英	205	0928205	2025/9/27	12948286	33.33	荧光共振能量转移法
董万财	101	0929101	2025/9/28	11201156	13.28	荧光共振能量转移法
何青贵	101	1001101	2025/9/30	12903358	26.72	荧光共振能量转移法
刘凤忱	107	1002107	2025/10/1	12947189	42.16	荧光共振能量转移法
李成柏	118	1002118	2025/10/1	12949376	13.65	荧光共振能量转移法
孙尚池	112	1007112	2025/10/6	12950115	22.59	荧光共振能量转移法
马乃玉	319	1008319	2025/10/7	12887522	38.91	荧光共振能量转移法
周奎琴	201	1008201	2025/10/7	12950570	28.37	荧光共振能量转移法

广州知力医学检验实验室
报告专用章

徐淑琴	115	1008115	2025/10/7	12950705	38.94	荧光共振能量转移法
陈德国	305	251014305	2025/10/13	12953266	73.73	荧光共振能量转移法
仲卫杰	215	20251015215	2025/10/14	12954308	28.87	荧光共振能量转移法
顾连春	202	1016202	2025/10/15	11838592	16.33	荧光共振能量转移法
沙喜贤	305	1018305	2025/10/17	12955582	4.346	荧光共振能量转移法
张永东	304	1019304	2025/10/18	12587325	33.58	荧光共振能量转移法
邱桂杰	217	1019217	2025/10/18	12945190	15.12	荧光共振能量转移法
金光俊	205	20251020205	2025/10/19	12955959	28.17	荧光共振能量转移法
高元力	201	1021201	2025/10/20	12956302	39.68	荧光共振能量转移法

报告专用章

实验仪器：荧光酶标仪　　　　品牌型号：赛默飞 374
