# Supplementary material for: Prognostic value of von Willebrand factor and ADAMTS-13 in patients with sepsis-induced coagulopathy
Source: Res Pract Thromb Haemost. 2026 Jul 14;10(5):106843. doi: 10.1016/j.rpth.2026.106843 (PMC13448020; doi:10.1016/j.rpth.2026.106843)
Supplement: Supplementary file 2 [file mmc2.pdf]

委托单位	吉林大学第一医院		
委托人	张凤	联系方式	155 3363 1069
被委托单位	同十生物医学（长春）有限公司		
项目负责人	免疫实验室	联系方式	18704316736

ADAMTS 13 抗原检测报告（共 240 例）

报告日期：2025-11-04

实验结果汇总：

姓名	床号	EP 管编号	入科时间	住院号	抗原结果（ng/ml）	方法学
任玉珍	311	0225311	2025/2/24	12838011	444	ELISA
肖庆彬	319	0224319	2025/2/24	12825560	490	ELISA
刘长宽	306	0227306	2025/2/26	11636562	283	ELISA
周玉珍	104	0227104	2025/2/26	12848119	421	ELISA
苏凤琴	302	0227302	2025/2/26	12848467	472	ELISA
吕春珍	106	0226106	2025/2/26	12848542	523	ELISA

战玉娥	303	0227303	2025/2/27	12848543	738	ELISA
王丽梅	121	0302121	2025/3/1	12849569	603	ELISA
侯美荣	102	0302102	2025/3/2	12849758	651	ELISA
马文忠	305	0306305	2025/3/5	12851241	241	ELISA
刘金英	202	0308202	2025/3/7	11620705	1010	ELISA
李淑英	201	0308201	2025/3/7	12852548	198	ELISA
张志武	221	0312221	2025/3/11	12342603	865	ELISA
文明	210	0312210	2025/3/11	12854776	473	ELISA
陈先超	103	0313103	2025/3/12	12854823	387	ELISA
冯桂杰	206	0316206	2025/3/15	12856368	1265	ELISA
张秀珍	305	0317305	2025/3/16	12803400	374	ELISA
代国才	320	250319j320	2025/3/18	12857738	403	ELISA
赵志兰	208	0319208	2025/3/19	12805358	345	ELISA
张桂兰	107	0321107	2025/3/20	11822152	769	ELISA
杜善民	306	0321306	2025/3/20	12858822	457	ELISA
许凤芹	211	0320211	2025/3/20	12858873	256	ELISA
马艳霞	307	0322307	2025/3/21	12807945	230	ELISA
苏中英	115	0324115	2025/3/21	12859792	312	ELISA
宋多军	320	250324320	2025/3/22	12860093	441	ELISA
高军	319	0325319	2025/3/24	12365545	923	ELISA
季生发	320	250325320	2025/3/25	12855501	782	ELISA
张淼	220	0327220	2025/3/26	12675458	668	ELISA
李斌	304	0327304	2025/3/26	12860040	346	ELISA
张永芬	306	0327306	2025/3/26	12862266	115	ELISA
邢永海	221	0328221	2025/3/27	12862602	329	ELISA

李芳	102	0329102	2025/3/28	12863199	467	ELISA
双喜	221	0331221	2025/3/29	12863456	326	ELISA
沈洪学	212	0331212	2025/3/30	12863765	302	ELISA
齐志红	320	0402J320	2025/4/1	12863253	500	ELISA
王春华	218	0402218	2025/4/1	12865003	337	ELISA
王维岐	112	0402112	2025/4/2	12399740	305	ELISA
康志学	203	0404203	2025/4/3	11971147	645	ELISA
陈玉环	307	0406307	2025/4/4	12862974	349	ELISA
腾凤香	110	0406110	2025/4/4	12864547	417	ELISA
张连海	203	0406203	2025/4/4	12866035	524	ELISA
隋素花	306	250409306	2025/4/7	12859534	497	ELISA
郭志才	201	0411201	2025/4/10	12868683	458	ELISA
王学政	218	0413218	2025/4/11	11048821	352	ELISA
宋希贵	205	0411205	2025/4/11	12864736	278	ELISA
窦中花	319	0413319	2025/4/11	12869485	422	ELISA
赵志国	115	0415115	2025/4/13	12364666	289	ELISA
何贵军	220	0413220	2025/4/13	12825413	329	ELISA
张生	311	0416311	2025/4/15	00017501	496	ELISA
衡国付	317	0416317	2025/4/15	12871359	476	ELISA
陈石	303	0416303	2025/4/15	12871561	436	ELISA
马孝臣	208	0417208	2025/4/16	12869203	198	ELISA
马晓艳	113	0416113	2025/4/16	12871403	264	ELISA
李淑霞	207	0419207	2025/4/17	12196143	614	ELISA
孙洪财	305	0421305	2025/4/19	12873043	253	ELISA
王玉艳	203	0421203	2025/4/21	12873722	434	ELISA

王俊平	308	0421308	2025/4/21	12874212	806	ELISA
包宝喜	311	0424311	2025/4/23	12875228	539	ELISA
刘海莉	305	0425305	2025/4/24	12833547	559	ELISA
唐玉祥	305	0426305	2025/4/25	12876477	223	ELISA
王桂荣	302	0428302	2025/4/27	11666519	302	ELISA
兰桂芹	111	0429111	2025/4/27	12874823	549	ELISA
付红光	313	0428313	2025/4/27	12877298	792	ELISA
汪秀芬	205	0430205	2025/4/29	12875871	549	ELISA
陈喜任	202	0430202	2025/4/30	12878178	568	ELISA
哈斯	218	0501218	2025/4/30	12878538	876	ELISA
刘玉杰	208	0502208	2025/5/1	12878589	326	ELISA
张士芹	117	0502117	2025/5/1	12878590	828	ELISA
王轩博	320	0504320	2025/5/3	12879008	219	ELISA
张凤梅	310	0505310	2025/5/4	12879372	227	ELISA
王锡富	109	0506109	2025/5/5	12879689	458	ELISA
王全	311	0506311	2025/5/5	12879403	841	ELISA
王金	203	0506203	2025/5/6	12879808	439	ELISA
田绍金	221	0507221	2025/5/6	12879852	616	ELISA
高淑琴	205	0508205	2025/5/7	12880626	400	ELISA
郭学会	108	0508108	2025/5/7	12881101	492	ELISA
段友发	202	0508202	2025/5/7	12881195	222	ELISA
王联海	111	0509111	2025/5/8	11472578	497	ELISA
王杰	101	0509101	2025/5/8	12881230	1125	ELISA
卢占军	203	0510203	2025/5/9	12881233	477	ELISA
戴永贵	220	0510220	2025/5/9	12881739	344	ELISA

于国平	221	0512221	2025/5/11	11590052	732	ELISA
于洪福	102	0512102	2025/5/11	12715603	566	ELISA
王凯	218	0512218	2025/5/11	12882480	675	ELISA
张丽华	204	0512204	2025/5/11	12882766	554	ELISA
潘立财	319	0514319	2025/5/13	12884187	643	ELISA
张春华	106	0514106	2025/5/14	12875377	70	ELISA
张凤传	119	0516119	2025/5/15	11605382	454	ELISA
李殿军	315	0515315	2025/5/15	12884724	597	ELISA
乔艳春	121	0516121	2025/5/15	12885141	1606	ELISA
唐连仲	302	0517302	2025/5/16	12451113	434	ELISA
高晓阳	318	0517318	2025/5/16	12884730	566	ELISA
李志芹	313	0517313	2025/5/16	12885428	490	ELISA
王玉兰	219	250519219	2025/5/17	12832366	219	ELISA
李志刚	220	250519220	2025/5/18	12885968	93	ELISA
张广才	318	250520318 张广才	2025/5/19	12881730	617	ELISA
李淑华	207	0520207	2025/5/19	12886596	400	ELISA
郝向文	215	0521215	2025/5/20	11355935	264	ELISA
张宇	111	0522111	2025/5/22	12887186	922	ELISA
张玉梅	208	0522208	2025/5/22	12888407	725	ELISA
董岩	221	0522221	2025/5/22	12888407	475	ELISA
柳树文	113	0523113	2025/5/23	12628820	626	ELISA
王际广	320	0523320	2025/5/23	12765873	591	ELISA
温希君	102	0523102	2025/5/23	12889013	491	ELISA
曲冬梅	319	20250528319	2025/5/27	12890343	760	ELISA
刘旭东	117	250530117	2025/5/28	12731155	374	ELISA

王延平	103	250529103	2025/5/28	12872844	689	ELISA
付书英	303	250529303	2025/5/28	12890975	447	ELISA
张万春	306	0530306	2025/5/29	12891310	473	ELISA
何亚娇	212	0530212	2025/5/29	12891556	437	ELISA
王清春	218	0601218	2025/5/31	12892064	401	ELISA
代红伟	208	0603208	2025/6/1	12886312	558	ELISA
郭庆文	209	0603209	2025/6/1	12892001	377	ELISA
徐亚芬	102	0601102	2025/6/1	12892175	704	ELISA
张志元	106	250603106	2025/6/1	12892458	449	ELISA
李凤云	304	20250606304	2025/6/4	12893859	292	ELISA
王旭红	313	20250606313	2025/6/5	12891473	262	ELISA
孙淑英	104	0606104	2025/6/5	12893088	369	ELISA
张群力	306	20250606306	2025/6/5	12894263	569	ELISA
张培琪	103	0607103	2025/6/6	12886137	420	ELISA
许国君	113	0607113	2025/6/6	12894781	520	ELISA
王延平	116	20250609116	2025/6/7	12872844	163	ELISA
包宝龙	217	20250609217	2025/6/7	12895259	654	ELISA
张春安	117	20250609117	2025/6/7	12895331	381	ELISA
温凤江	219	250610219	2025/6/9	12895882	957	ELISA
郑宝石	315	250610315	2025/6/10	12896145	1097	ELISA
张宝义	306	0614306	2025/6/14	12898571	526	ELISA
马贵	118	0616118	2025/6/15	11773263	886	ELISA
王纪英	106	0616106	2025/6/15	12899237	1272	ELISA
逯胜金	107	0616107	2025/6/16	12899238	381	ELISA
张建明	208	250618208	2025/6/17	12900536	447	ELISA

刘忠山	306	0622306	2025/6/21	12902059	611	ELISA
王秀兰	218	0623218	2025/6/22	12902571	1470	ELISA
马启云	308	0624308	2025/6/23	12899970	208	ELISA
刘明友	316	0625316	2025/6/24	12903814	98	ELISA
祝福	319	0627319	2025/6/26	12585995	698	ELISA
刘凤芝	302	0627302	2025/6/26	12904319	359	ELISA
董书有	220	0628220	2025/6/27	12518227	670	ELISA
张耀魁	308	0628308	2025/6/27	12905156	918	ELISA
李成建	111	0629111	2025/6/28	12905416	418	ELISA
邹继刚	204	250701204	2025/6/30	12904202	789	ELISA
李永芹	202	250701202	2025/6/30	12905744	302	ELISA
李桂珍	319	0701319	2025/7/1	12906487	1090	ELISA
于珍	206	20250703206	2025/7/2	12907094	529	ELISA
尹志强	216	20250703216	2025/7/2	12907141	846	ELISA
韩杰	115	250704115	2025/7/3	12255216	961	ELISA
柴永波	309	250704309	2025/7/3	12908078	957	ELISA
包力德日高	318	0706318	2025/7/5	12908480	591	ELISA
刘洪岩	202	0707202	2025/7/6	12908829	277	ELISA
姜春有	117	0708117	2025/7/7	12423866	608	ELISA
韩凤侠	110	0709110	2025/7/8	12847711	445	ELISA
付玉清	112	20250709112	2025/7/8	12909847	419	ELISA
刘福芹	201	20250710201	2025/7/9	12822280	318	ELISA
杨国发	120	0711120	2025/7/10	12911290	594	ELISA
赵文江	306	0714306	2025/7/13	12892753	804	ELISA
赵洪丽	118	20250715118	2025/7/14	12912389	666	ELISA

尹凤云	310	250716310	2025/7/15	12913342	807	ELISA
张少华	302	0717302	2025/7/16	12914329	730	ELISA
陆玉华	320	0718320	2025/7/17	12914776	425	ELISA
王守文	304	0720304	2025/7/19	12915380	471	ELISA
谢学勤	221	0721221	2025/7/20	12915511	690	ELISA
董林	306	250722306	2025/7/21	12916377	589	ELISA
张兴龙	207	250722207	2025/7/21	12916599	902	ELISA
荆纪元	320	20250724303	2025/7/23	11486900	1027	ELISA
连桂芝	221	250725221	2025/7/24	12916291	621	ELISA
杨继华	101	0725101	2025/7/24	12918213	706	ELISA
谢井玲	108	0726108	2025/7/25	12918606	506	ELISA
杨永臣	112	0727112	2025/7/26	12404647	371	ELISA
徐忠	105	0428111-1	2025/7/26	12918594	572	ELISA
朱艳森	113	250729113	2025/7/28	12918920	538	ELISA
王淑伟	208	0803208	2025/8/2	12921962	280	ELISA
李淑艳	301	2025804301	2025/8/3	12916032	871	ELISA
谢跃武	117	250805117	2025/8/4	12834442	703	ELISA
刘书琴	221	250805221	2025/8/4	12922581	773	ELISA
刘树仁	111	20250806111	2025/8/5	12679536	647	ELISA
吴志	308	20250806308	2025/8/5	12923349	847	ELISA
李淑琴	303	0802303	2025/8/7	12923812	758	ELISA
单洪光	304	0810304	2025/8/9	11621050	853	ELISA
李福春	101	0810101	2025/8/9	12924923	757	ELISA
马玉芹	306	250812306	2025/8/11	12872533	571	ELISA
陈淑清	209	250812209	2025/8/11	12926006	912	ELISA

潘丽君	119	250812119	2025/8/11	12926729	492	ELISA
邵柱文	221	0813221	2025/8/12	12926734	619	ELISA
贾香阁	221	250815221	2025/8/14	11303331	351	ELISA
杨威山	304	0817304	2025/8/15	12923855	1768	ELISA
魏道行	221	0816221	2025/8/16	12928796	384	ELISA
张洪国	221	0817221	2025/8/17	12929069	1084	ELISA
杨艳芳	102	0820102	2025/8/19	12930563	495	ELISA
李淑艳	219	0820219	2025/8/19	12930617	416	ELISA
李桂荣	315	0821315	2025/8/20	12914072	280	ELISA
王希江	202	20250821202	2025/8/20	12930639	849	ELISA
王永强	218	0822218	2025/8/21	12929734	745	ELISA
何显峰	209	0824209	2025/8/23	12929045	1552	ELISA
高代岭	202	0824202	2025/8/23	12932055	1091	ELISA
王献勤	208	0824208	2025/8/23	12932229	1282	ELISA
李树元	304	0824304	2025/8/23	12932252	959	ELISA
徐殿玉	204	0825304	2025/8/24	12565665	689	ELISA
陈书媛	103	20250827103	2025/8/26	12931448	958	ELISA
高志荣	120	20250828120	2025/8/27	12324945	260	ELISA
鲁占山	316	20250828316	2025/8/27	12933924	441	ELISA
刘淑香	217	20250828217	2025/8/27	12933928	351	ELISA
于海林	220	0831220	2025/8/30	12935322	216	ELISA
霍荣华	104	0831104	2025/8/30	12935559	367	ELISA
李立明	311	0831311	2025/8/31	12935580	900	ELISA
王艳波	303	250902303	2025/9/1	12936352	1023	ELISA
王志萍	319	20250904319	2025/9/3	12937516	481	ELISA

刘桂艳	209	0905209	2025/9/4	12933031	336	ELISA
董昕卓	306	0905306	2025/9/5	00806280	188	ELISA
刘会君	109	0906109	2025/9/5	12345468	664	ELISA
汪佳荣	304	0906304	2025/9/5	12938244	898	ELISA
刘书真	320	0909320	2025/9/8	12939003	516	ELISA
邱琬玥	113	0909113	2025/9/8	12939647	1117	ELISA
高雨林	108	20250910108	2025/9/9	12897064	507	ELISA
邹万发	111	0914111	2025/9/13	12941576	434	ELISA
张兰	117	0914117	2025/9/13	12941808	439	ELISA
吴会娟	206	0917206	2025/9/16	12942142	595	ELISA
孙中芹	212	0917212	2025/9/16	12943187	418	ELISA
李龙蛟	101	0919101	2025/9/19	12944405	360	ELISA
李凤	306	2025922306	2025/9/21	11800557	1090	ELISA
宋庆	202	250923202	2025/9/22	12945363	493	ELISA
耿玉林	319	20250925319	2025/9/24	12946630	816	ELISA
朱京里	110	250926110	2025/9/25	12947570	1350	ELISA
李金	218	0928218	2025/9/27	12937747	671	ELISA
姜连英	205	0928205	2025/9/27	12948286	772	ELISA
董万财	101	0929101	2025/9/28	11201156	591	ELISA
何青贵	101	1001101	2025/9/30	12903358	514	ELISA
刘凤忱	107	1002107	2025/10/1	12947189	864	ELISA
李成柏	118	1002118	2025/10/1	12949376	222	ELISA
孙尚池	112	1007112	2025/10/6	12950115	391	ELISA
马乃玉	319	1008319	2025/10/7	12887522	642	ELISA
周奎琴	201	1008201	2025/10/7	12950570	913	ELISA

徐淑琴	115	1008115	2025/10/7	12950705	666	ELISA
陈德国	305	251014305	2025/10/13	12953266	721	ELISA
仲卫杰	215	20251015215	2025/10/14	12954308	642	ELISA
顾连春	202	1016202	2025/10/15	11838592	271	ELISA
沙喜贤	305	1018305	2025/10/17	12955582	258	ELISA
张永东	304	1019304	2025/10/18	12587325	308	ELISA
邱桂杰	217	1019217	2025/10/18	12945190	309	ELISA
金光俊	205	20251020205	2025/10/19	12955959	823	ELISA
高元力	201	1021201	2025/10/20	12956302	653	ELISA

实验仪器：酶标仪（型号，品牌）：Epoch2 ，Bio-tek
